# The cost-effectiveness of screening tools used in the diagnosis of fetal alcohol spectrum disorder: a modelled analysis

**DOI:** 10.1186/s12889-019-8110-5

**Published:** 2019-12-27

**Authors:** Patrick Berrigan, Gail Andrew, James N. Reynolds, Jennifer D. Zwicker

**Affiliations:** 1 School of Public Policy, University of Calgary, 906 8th Avenue SW, 5th Floor, Calgary, Alberta T2P 1H9 Canada; 2 Faculty of Medicine and Dentistry, University of Alberta, Edmonton Clinic Health Academy, 5th Floor, 11405 - 87 Avenue NW, Edmonton, Alberta T6G 1C9 Canada; 3 Glenrose FASD Clinic, Alberta Health Services, 10230 111 Avenue NW, Edmonton, Alberta T5G 0B7 Canada; 4 Department of Biomedical and Molecular Sciences, Queen’s University, Botterell Hall, Room 563, 18 Stuart Street, Kingston, Ontario K7L 3N6 Canada; 5 Faculty of Kinesiology, University of Calgary, 2500 University Drive NW, Calgary, Alberta T2N 1N4 Canada

**Keywords:** Cost-effectiveness analysis, Fetal alcohol spectrum disorder, Screening

## Abstract

**Background:**

Fetal Alcohol Spectrum Disorder (FASD) is characterized by physical and neurological abnormalities resulting from prenatal alcohol exposure. Though diagnosis may help improve patient outcomes, the diagnostic process can be costly. Subsequently, screening children suspected of FASD prior to diagnostic testing has been suggested, to avoid administering testing to children who are unlikely to receive a diagnosis. The present study set out to assess the cost-effectiveness of currently recommended FASD screening tools.

**Methods:**

The screenings tools evaluated were chosen from Children’s Healthcare Canada’s National Screening Toolkit for Children and Youth Identified and Potentially Affected by FASD and include meconium testing of fatty acid ethyl esters (meconium testing) and the neurobehavioral screening tool (NST). An economic model was constructed to assess cost-effectiveness. One-way and probabilistic sensitivity analyses were conducted to assess the robustness of findings. Costs reflect 2017 Canadian dollars and the perspective is the public healthcare system.

**Results:**

Both screening tools evaluated resulted in reduced costs and fewer diagnosed years of life than a no screening strategy in which all children suspected of FASD receive diagnostic testing. The model predicts that screening newborns with meconium testing results in a reduced cost of $89,186 per 100 individuals screened and 38 fewer diagnosed years of life by age 18, corresponding to an incremental cost-effectiveness ratio (ICER) of $2359. Screening children with the NST resulted in a reduced cost of $183,895 per 100 individuals screened and 77 fewer diagnosed years of life by age 18, corresponding to an ICER of $2390.

**Conclusion:**

Findings suggest that screening is associated with less use of healthcare recourses but also fewer years of life with an FASD diagnosis over a no screening strategy. Since diagnosis can be key to children receiving timely and appropriate health and educational services, cost-savings must be weighed against the fewer years of life with a diagnosis associated with screening.

## Background

Fetal Alcohol Spectrum Disorder (FASD) is characterized by physical and neurological abnormalities that result from prenatal exposure to alcohol. Common symptoms associated with FASD include facial dysmorphia, stunted growth, abnormal neurodevelopment, behavioral issues, and impairments to cognitive function [1, 2]. The prevalence of FASD in Canada is estimated to be between 2 and 3% and individuals impacted by FASD often face substantial burden [3]. Evidence suggests that this population experiences lower rates of academic achievement, higher rates of incarceration, increased risk of problematic substance use, and higher overall mortality than the general public [4–7]. In addition to significant impacts to patients’ wellbeing, the annual economic burden of FASD to society is large and was estimated to be $1.9 billion in 2017 Canadian Dollars [8].

Standard care for FASD typically focuses on individualized symptoms management and evidence suggests that early intervention can be beneficial [9]. An FASD diagnosis can allow service providers to better anticipate disabilities associated with FASD, lead to more timely treatments, and can allow for better access to services [10, 11]. However, accurate diagnosis can be difficult and resource intensive to obtain. Typically, diagnosis requires a substantial battery of testing conducted by a range of specialized healthcare professionals costing up to $5000 in 2017 Canadian Dollars [12]. Screening children suspected of FASD prior to diagnostic testing has been suggested, as a method to avoid administering testing to children who are unlikely to receive diagnoses [13]. However, screening is not without its limitations, as screening tools do not have perfect accuracy, screening may result in children who would test positive for FASD not being recommended to receive diagnostic testing.

As a result, the present study constructed an economic model to assess the value for money of tools used to screen children suspected of FASD prior to diagnostic testing. Specifically, this study measures the incremental cost associated with an additional year of life with an accurate FASD diagnosis.

Despite the substantial burden, few studies have evaluated the cost-effectiveness of medical procedures associated with FASD. Thanh et al. [14] conducted a modelled cost-effectiveness analysis (CEA) of an intervention aimed at preventing FASD by providing case management support to mothers. Hopkins et al. [15] conducted a modelled CEA of universal versus targeted screening of newborns for FASD. To the best of our knowledge, the present study is the first to assess the cost-effectiveness of screening tools in FASD.

The screening tools included in the present study were chosen from the Canadian Association of Pediatric Health Center (CAPHC) National Screening Toolkit for Children and Youth Identified and Potentially Affected by FASD. CAPHC, now referred to as Children’s Healthcare Canada, is a national network that provides guidance on best practice in pediatric care. CAPHC’s toolkit is comprised of: i) the Neurobehavioral Screening Tool (NST); ii) meconium fatty acid ethyl esters (FAEE) testing (meconium testing); iii) the Maternal Drinking Guide Tool; iv) the Medicine Wheel Student Index/Medicine Wheel Developmental History (Medicine Wheel); and v) FASD Screening & Referral Form for Youth Probation Officers (Asante Screening Tool) [13, 16].

As the cost-effectiveness of screening tools for FASD is dependent on a variety of situational factors, it is not possible that a single tool from the CAPHC toolkit is optimal in all situations with respect to cost-effectiveness. For example, meconium testing is the only screening tool in the toolkit that is applicable for newborns when confirmation of alcohol exposure cannot be established directly from caregivers. Subsequently, the present study did not set out to identify a single most cost-effective tool but rather to assess the cost-effectiveness of the tools under reasonable scenarios.

## Methods

### Screening tools

Screening tools were chosen from the CAPHC toolkit for inclusion based on two criteria: i) the cost of administering the screening tool was available or a reasonable approximation could be estimated and ii) an estimate of the diagnostic accuracy, referring to the sensitivity and specificity of the screening tool to FASD, was available or a reasonable approximation could be estimated. Sufficient information on the diagnostic accuracy of the Maternal Drinking Guide Tool, the Medicine Wheel, and the Asante Screening Tool was not identified.

Information was identified to assess the cost-effectiveness of screening tools for two scenarios. The first compares *screening newborns suspected of FASD* via *meconium testing prior to diagnostic testing* versus *no screening but diagnostic testing for all newborns suspected FASD*. Meconium testing screens fecal matter that accumulates over the second and third trimesters for chemical signatures of ethanol that can be indicative of prenatal alcohol exposure [17]. The second compares *screening 5 year olds with the NST prior to diagnostic testing* versus *no screening but diagnostic testing for all children suspected of FASD*. The NST is a questionnaire that asks caregivers about their child’s FASD associated behaviors and risk factors. Depending on the number of positive responses, the questionnaire recommends diagnostic testing or no diagnostic testing [18].

The present study does not directly compare the cost-effectiveness of meconium testing to the NST as these tools are not directly comparable due to the age groups for which they are intended. Meconium testing is for newborn populations and the NST is meant to be used in school age populations.

### Cost-effectiveness analysis

In the present study, incremental cost-effectiveness ratios (ICER) are used to assess value for money [19]. ICER are calculated by dividing the difference in cost by the difference in effectiveness between two interventions. To assess the value for money of an intervention relative to another, decision-makers can compare ICER values to the amount their jurisdiction would be willing to pay (WTP) to gain or willing to accept (WTA) to forgo the effectiveness outcome included in the ICER. Costs are expressed in 2017 Canadian dollars and reflect the perspective of the public healthcare payer. To calculate ICER, a Markov model was constructed. Model parameters such as costs and estimates of the accuracy of screening tools were informed using published literature, expert opinion, and in some cases assumptions based on approaches previously undertaken within the literature.

### Model

A hypothetical cohort of children suspected of FASD are evaluated using two versions of an economic model: i) compares screening with meconium testing to a no screening strategy where all children suspected of FASD receive diagnostic testing and ii) compares screening with the NST to a no screening strategy where all children suspected of FASD receive diagnostic testing. Using the model, the total cost and the number of years with an accurate FASD diagnosis were tracked for each strategy until children reached 18 years of age and these values were used to calculate ICER.

Though quality adjusted life years (QALY) are commonly recommended for use as the primary outcome measure in economic evaluations [19], at present the impact of an FASD diagnosis on patients’ health related quality of life (HRQoL) is not understood [20]. As a substitute to QALY, the number of diagnoses or other diagnoses based outcomes are sometimes used as a primary effectiveness measure in CEA of screening strategies [21]. This study uses years with a diagnosis instead of the number of diagnoses, as the former better reflects the temporal nature of a diagnosis. A diagnosis happens at single point in time but has long-term implications. The discounted present value of benefits using the dynamic outcome of years with an accurate FASD diagnosis better accounts for the temporal nature of the decision problem.

**Fig. 1:**
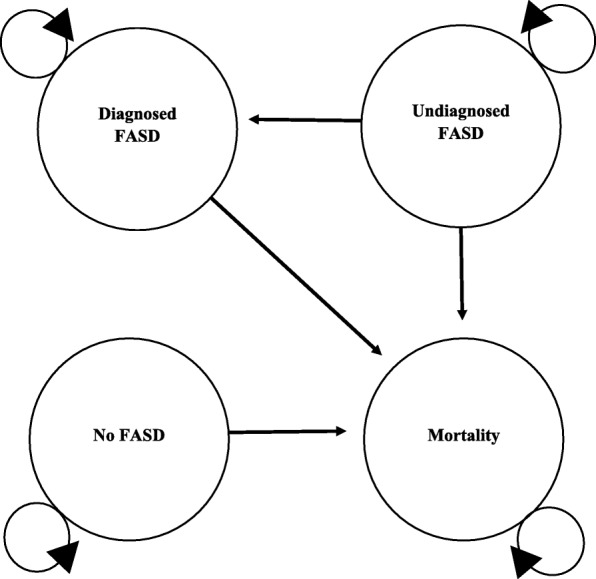
Markov Diagram

**Fig. 2:**
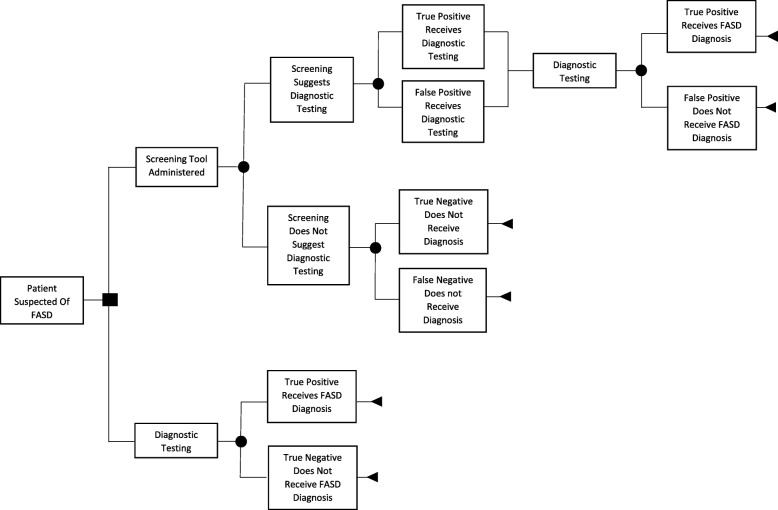
Decision Tree Informing Initial Distribution of Cohort between States

In the model, screening can result in true/false positives and patients receive diagnostic testing or true/false negatives and patients do not receive diagnostic testing. Diagnostic testing in both the screening and no screening strategies can result in an FASD diagnosis or no FASD diagnosis. Since newborns who screen positive for prenatal alcohol exposure via meconium testing do not often receive diagnostic testing immediately, the model applied a five-year lag between positive screen with meconium testing and patients receiving diagnostic testing. Figure 1 shows the Markov diagram for the model and Fig. 2 shows the decision tree that informed the initial distribution of the hypothetical cohort between states.

In the model, screening can influence costs by reducing the number of individuals who go on to receive diagnostic testing and by affecting the ratio of diagnosed to undiagnosed patients, which are assumed to have different costs and mortality. The study assumes that a year of life with a diagnosis is associated with better outcomes for patients with FASD than a year of life without a diagnosis [1, 22]. A half-cycle correction was applied. The half cycle correction is applied so that patients’ transition through Markov states reflects a mid cycle transition versus at either the beginning or end of the Markov cycle [23].

### Model parameters

#### Hypothetical cohort

The present study assumes a hypothetical cohort of 100 children of which 66% (SD = 1.4%) meet the criteria for FASD. This represents the percentage of children who received an FASD diagnosis after receiving consultation for suspected FASD at specialized clinics in Western Canada in 2005 [24]. The cohort was assumed to consist of 50% females. This assumption was justified based on the most recent study within Canada on FASD prevalence, which showed similar rates of FASD between males and females [3].

### Diagnostic accuracy of screening tools

#### Meconium testing

Only studies that included newborns with alcohol exposures at any time during gestation were considered for inclusion, as some identified studies reported newborns with alcohol exposures in the second and third trimesters only. Such studies would inflate the diagnostic accuracy of meconium testing by censoring first trimester alcohol exposures, which can be the most impactful, that meconium testing will not detect. If studies reported multiple criteria for a positive screen on the same group of patients, the strategy with the highest sensitivity was incorporated in the analysis. This is based on CAPHC’s assertion that screening tools should be liberal in their selection for diagnostic testing. From the findings of a recent systematic review [17], four relevant studies were identified [25–28] (Table 1). It should be noted that identified studies reported the diagnostic accuracy of meconium testing to prenatal alcohol exposure and not positive FASD diagnoses. Study results were pooled using a random effects approach [29]. The mean sensitivity was 92.4% (SD = 8.1%) and the mean specificity was 51.5% (SD = 19.7%).

**Table 1:** Diagnostic Accuracy of Meconium Testing

Study	Study Information	Criteria for Positive Screen	Sensitivity (SD)^a^	Specificity (SD)^a^
1. Bakhireva et al., 2014 [25]	Sample Size = 60 Positive Cases Included = 28 Positive Cases = ≥ 0.21 oz. alcohol/day at enrollment or ≥ 2.0 oz. of alcohol/drinking day. Controls = No binge drinking in the periconceptional period; ≤ 0.14 oz. alcohol/day in periconceptional period; and no drinking at enrollment. FAEEs Tested = Ethyl Palmitate, Ethyl Stearate, Ethyl Oleate, Ethyl Linoleate. Limit of Detection = 50 ng/g	> 600 ng/g all four FAEEs to meconium.	100% ^b^ (1.9%)	13% (5.9%)
2. Ostrea et al., 2006 [26]	Sample Size = 124 Positives Cases Included = 93 Positive Cases = Mothers who used alcohol at the time of conception and/or any time during pregnancy. Controls = Mothers who reported no alcohol intake around the time of conception or in pregnancy. FAEEs Tested = Ethyl Myristate Limit of detection = 50 ng/g	> 50 ng/g ethyl myristate to meconium.	68% ^c^ (4.8%)	29% ^c^ (8.0%)
3. Bearer et al., 2003 [27]	Sample Size = 27 Positives Cases Included = 21 Positive Cases = ≥ 1.0 oz. alcohol/day or ≥ 2 incidents of binge drinking/month in the first trimester of pregnancy. Controls = Mothers who abstained from drinking during pregnancy. FAEEs Tested = Ethyl Oleate Limit of Detection = NA	> 13 ng/g ethyl oleate to meconium.	100% ^b^ (2.1%)	67% (17.8%)
4. Chan et al., 2003 [28]	Sample Size = 200 Positive Cases Included = 17 Positive Cases = Mothers who reported any drinking in pregnancy. Controls = Mothers who reported no drinking in pregnancy. FAEEs Tested = Ethyl Palmitate, Ethyl Stearate, Ethyl Oleate, Ethyl Linoleate. Limit of Detection = 50 ng/g	> 600 ng/g all four FAEEs to meconium. ^c^	100% ^b^ (2.3%)	98% (1.0%)

^a^ If SD were not reported, they were calculated using the beta distribution variance formula

^b^ Sensitivity and specificity were assumed to be 99% instead of 100%, as the beta distribution calculates a variance of 0 for mean values of 100%

^c^ Estimates were taken from a systematic review and not reported in the corresponding study

### The NST

As with meconium testing, if studies reported multiple criteria for positive screens on the same group of patients, the strategy with the highest sensitivity was incorporated in the analysis. From the findings of a recent systematic review [18], four studies reporting the diagnostic accuracy of the NST were identified [30–33] (Table 2). Study results were pooled using a random effects approach [29]. The mean sensitivity was 85.9% (SD = 5.5%) and the mean specificity was 72.9% (SD = 10.7%).

**Table 2:** Diagnostic Accuracy of the Neurobehavioral Screening Tool

Study	Study Information	Criteria for Positive Screen	Sensitivity (SD)^a^	Specificity (SD) ^a^
1. LaFrance et al., 2014 [30]	Sample Size = 80 Positives Included = 48 Positive Cases = Children with FASD diagnosis. Controls = Typically developing children. Average Age = 12	≥ 6 of items 1–7 or ≥ 3 of items 1–4.	63% (6.9%)	100% ^b^ (1.7%)
2. Breiner et al., 2013 [31]	Sample Size = 60 Positives Included = 17 Positive Cases = Children with FASD diagnosis. Controls = 18 children suspected for FASD but for whom diagnosis could not be confirmed and 25 typically developing children. Median Age = 5 ^c^	≥ 5 of items 1, 2, 4–8.	94% (5.6%)	96% (3.0%)
3. Nash et al., 2011 [32]	Sample Size = 109 Positives Included = 56 Positive Cases = Children with FASD diagnosis. Controls = Typically developing children. Average Age = 10 Sample Size = 106	≥ 3 of items 1–10	98% (1.9%)	42% (6.7%)
	Positives Included = 56 Positive Cases = Children with FASD diagnosis. Controls = Children with ADHD diagnosis. Average Age = 10	≥ 2 of items 1, 4, 8, 9, 10.	89% (4.1%)	42% (6.9%)
4. Nash et al., 2006 [33]	Sample Size = 60 Positives Included = 30 Positive Cases = Children with FASD diagnosis. Controls = Typically developing children. Median Age = 11 ^c^	≥ 6 of items 1–7	86% (6.2%)	82% (6.9%)
	Sample Size = 60 Positives Included = 30 Positive Cases = Children with FASD diagnosis. Controls = Children with ADHD diagnosis. Median Age = 11 ^c^	≥ 3 of items 1, 4, 8, 9, 10.	81% (7.0%)	72% (8.1%)

^a^ If SD were not reported, they were calculated using the beta distribution variance formula

^b^ Sensitivity and specificity were assumed to be 99% instead of 100%, as the beta distribution calculates an SD of 0 for mean values of 100%

^c^ If the average age of study participants was not provided, the median was reported

### Accuracy of diagnostic testing

The present study assumes perfect accuracy for diagnostic testing. As it is likely that missed diagnosis of FASD occur, this assumption is assessed in one-way sensitivity analysis. Assuming perfect diagnostic accuracy of diagnostic testing has been undertaken previously in the literature when the sensitivity and/or specificity of a diagnostic test is not known [21]. A benefit of this approach is that it applies a best-case scenario for the accuracy of diagnostic testing in the model. This can help contextualized sensitivity analysis surrounding the parameter. In the present study, this assumption has the effect of biasing results against the screening strategies.

### Cost of screening tools

#### Meconium testing

A cost of $175 was used to approximate the cost of meconium testing [15]. This value was based on the price of meconium testing charged to patients at the Hospital for Sick Children (Toronto, Ontario, Canada) and taken directly from a previous study Hopkins et al. [15].

### The neurobehavioral screening tool

To estimate the cost of administering the NST, the present study included time spent interacting with caregivers, time required to administer the NST, and an estimate of the cost of relevant overhead (office supplies, printing services, technology etc.). This included 15 min of a social worker’s time, 7.0 min of a psychologist’s time, and $5.00 in overhead costs. This corresponded to an estimate of $20 per NST administered. The cost of health providers’ time was based on reimbursement within Ontario, Canada.

#### Cost of diagnostic testing

The cost of diagnostic testing, which includes a physical examination, dysmorphology assessment, neurobehavioral assessment, and prenatal exposure to alcohol confirmation was estimated to be $3870 [12].

### Cost of health services use

#### First year of life

To approximate the annual cost of health service use by patients with FASD diagnoses, this study relies on the work of Stade et al. [34], who report societal costs for a group of patients of average age 12.9 years with FASD diagnoses. For the first year of life, Stade et al. [34] report a cost of $20,265 for health services spending. These costs reflect health service utilization related to managing early life medical complications associated with FASD such as low birth weight or prematurity. This cost was applied to the first year of life for the undiagnosed and no FASD groups as well.

### Diagnosed FASD

For all subsequent years, a cost of $4346 per year was applied to the diagnosed FASD population based on the work of Stade et al. [34]. This cost included doctor visits, hospitalizations, emergency department visits, medications, diagnostic tests, and medical devices [34].

### Undiagnosed FASD

A lack of information on the cost of healthcare service utilization for undiagnosed patients is often a limitation in CEA of screening strategies [21], as costing studies are not often undertaken in undiagnosed populations. As a result, CEA in screening strategies often need to make assumptions, to approximate costs for undiagnosed populations. To estimate the cost of undiagnosed FASD after the first year of life, this study combines the work of Stade et al. [34] and McLachlan et al. [35]. McLachlan et al. [35], conducted a chart review to investigate the medical, educational, and social services recommended to a group of 70 children assessed for FASD. Of these children: 45 received a diagnosis of FASD; nine had their diagnosis deferred; and FASD was not diagnosed in 16. A deferred diagnosis indicates that FASD could not be confirmed but the diagnostic team was unwilling to rule out FASD. Subsequently, future reassessment is recommended. Though not significant at standard levels (χ^2^ = 1.48; *p*-value = 0.223), McLachlan et al. [35] found that deferred children were 22.2% less likely to be recommended psychiatric treatment than children with a diagnosis. Assuming that service use associated with deferred patients reflects that of patients with undiagnosed FASD and combining this data with the cost reported in Stade et al. [34] for diagnosed FASD, results in an estimated annual cost of undiagnosed FASD of $3441 (For further details see Additional file 1). This assumption is tested in one-way sensitivity analysis. Research suggests that a majority portion of differed patients will go on to receive an FASD diagnosis at some point in their life [22].

### No FASD

Patients without FASD were assumed to use healthcare resources at a rate of $3101/year. This value was calculated using the same method as the cost for undiagnosed FASD [34, 35].

### Rate of future diagnosis

Patients with FASD who do not receive a diagnosis due to a false negative in screening are assumed to receive future diagnoses at a rate of 5% per year. At present, the rate of future diagnosis for patients with FASD who fail to receive a diagnosis because of a false negative in screening is not known. As a result, this parameter has been estimated based on the assessment of the present study’s authors. This assumption is assessed in one-way sensitivity analysis. These patients are assumed to receive repeated screening and diagnostic testing during subsequent diagnoses.

### Mortality

Based on the findings of a recent systematic review [36], one study has reported mortality in FASD [6]. Burd et al. [6] report a standardized mortality ratio (SMR) for a cohort of individuals diagnosed with FASD of 3.15. Mortality was assumed to be elevated 10% in the undiagnosed FASD population and to reflect that of diagnosed FASD in the no FASD population. At present, the rate of mortality for undiagnosed patients is not known and this parameter was informed based on the assessment of the present study’s authors. Mortality assumptions were assessed in one-way sensitivity analysis. SMR were combined with Statistics Canada life tables to estimate mortality.

### Discounting and time horizon

Cost and outcomes occurring beyond 1 year were discounted at a rate of 1.5% [19]. Discounting weights events occurring sooner to a greater extent than those occurring later to account for societal preference for the present. Costs and outcomes were aggregated until children reached age 18. This time-horizon was chosen as consultation with experts suggested that pediatric diagnosis is of greater value for improving patient outcomes than diagnosis in adulthood. Additionally, as there are few treatment options available for adults [9], it is not clear how service utilization between diagnosed and undiagnosed adults would differ.

### Probabilistic analysis

To conduct probabilistic analysis (PA), values were randomly sampled for each model parameter from a distribution and then used to calculate ICER. This process was repeated 5000 times using Microsoft Excel (Microsoft Corporation, Redmond, WA, USA).

For parameter values that represent percentages, a beta distribution was applied with mean and SD based on literature-derived estimates. Two exceptions to this are i) the rate of subsequent diagnoses was varied subject to a uniform distribution over the range 3 to 7% and ii) the mortality for undiagnosed FASD was varied subject to a uniform distribution by an increase of 0 to 20% relative to the mortality of diagnosed FASD. At present, uncertainty for the aforementioned parameters (i and ii) is not well understood, the authors of the present study chose these intervals to reflect a large degree of uncertainty for these parameters. The uniform distribution was chosen, as it makes each value within the PA interval equally likely further accounting for uncertainty. Cost for screening tools and diagnostic testing were varied subject to the normal distribution within the interval plus or minus 25% of the parameter value with an SD of 10% of the parameter value. Estimates for costs based on experimental results were varied subject to the log-normal distribution with an SD of 10% of the parameter value. Parameter values for mortality were varied subject to the normal distribution but it was assumed that mortality could not be superior to the general public. The SD for the mortality of patients with no FASD represents the SD of diagnosed FASD inflated by 1.25, to account for uncertainty regarding mortality rates in this population. Sensitivity and specificity estimates were not correlated in PA. This may result in the model overstating uncertainty. For a list of model parameters and distributional assumptions, see Table 3.

**Table 3:** Parameter Values, Standard Deviations, and Distributional Assumption

Parameter and Reference	Mean (SD)	Distributional Assumption
Hypothetical cohort characteristics		
% Positive cases [24]	66.3% (1.4%)	Beta
% Female	50.0%	Not varied
Age screened Meconium Testing	Birth	Not varied
Age screened NST	5 years	Not varied
Diagnostic accuracy of screening tools		
Meconium testing		
Sensitivity [25–28]	92.4% (8.1%)	Beta
Specificity [25–28]	51.5% (19.7%)	Beta
The NST		
Sensitivity [30–33]	85.9% (5.5%)	Beta
Specificity [30–33]	72.9% (10.7%)	Beta
Accuracy of Diagnostic Testing		
Sensitivity	100%	Not varied
Specificity	100%	Not varied
Cost of screening tools and Diagnostic Testing		
Meconium testing [15]	$175 ($18)	Normal (bounded ±25% of mean)
The NST ^b^	$20 ($2)	Normal (bounded ±25% of mean)
Cost of diagnostic testing [12]	$3870 ($387)	Normal (bounded ±25% of mean)
Annual Cost of Healthcare Service Use		
First year of life [34]	$15,976 ($1598)	Log-normal
Diagnosed FASD [34]	$3426 ($343)	Log-normal
Undiagnosed FASD [34, 35]	$2713	Varied based on inputs ^a^
Diagnosed recommended to receive psychiatric care [35]	55.6% (7.3%)	Beta
Undiagnosed recommended to receive psychiatric care [35]	33.0% (14.7%)	Beta
No FASD [34, 35]	$3101	Not varied
Future Diagnosis Rate		
Rate of future diagnosis for undiagnosed patients ^c^	5%	Uniform (bounded ±2%)
Mortality		
Diagnosed FASD [6]	3.15 (1.6)	Normal
Increased mortality for undiagnosed	10%	Uniform (bounded ±10%)
FASD relative to diagnosed ^c^		
No FASD^c^	3.15 (2.0)	Normal

The values for the Annual Cost of Healthcare Service Use in Table 3 reflect that prior to adjusting for inflation. The Annual Cost of Healthcare Service Use parameters were varied prior to adjusting for inflation and then inflated for probabilistic analysis

^a^ Inputs refer to Diagnosed recommended to receive psychiatric care and Undiagnosed recommended to receive psychiatric care

^b^ Parameter was informed with unpublished local data

^c^ Parameter was informed based on authors’ assumption

### One-way sensitivity analysis

To conduct one-way sensitivity analysis, parameter values for key model inputs were varied by plus and minus 25% of the parameter value. Parameters included in one-way analysis include the sensitivity and specificity of screening tools, the sensitivity of diagnostic testing, the number of positive cases in the cohort, the annual cost of diagnosed and undiagnosed FASD, the cost of diagnostic testing, the cost of screening tools, future diagnosis rates, and mortality rates. Alternative discount rates were assessed in scenario analysis based on the recommendation of CADTH [19].

### Results

### Cost-effectiveness analysis

The model predicts that screening newborns with meconium testing results in a cost savings of $89,186 per 100 individuals screened and approximately 38 fewer years of life with an FASD diagnoses over the no screening strategy corresponding to an ICER of $2359. Screening 5 year olds with the NST resulted in a cost savings of $183,895 per 100 individuals screened and approximately 77 fewer years of life with an FASD diagnoses over the no screening strategy corresponding to an ICER of $2390 (Table 4). Alternative discount rates of 0 and 3% did not substantially affect cost-effectiveness, resulting in ICER of $2256 and $2459 for meconium testing and $$2253 and $2531 respectively for the NST.

**Table 4:** Incremental Cost-effectiveness Ratios

Strategy	Cost	Effectiveness	Δ Cost	Δ Effectiveness	ICER
No screening	$7,007,542.41	709.34			
Meconium Testing	$6,918,356.37	671.54	-$89,186.04	−37.80	$2359.15
No screening	$4,366,538.94	778.23			
The NST	$4,182,643.95	701.28	-$183,894.98	−76.95	$2389.86

Cost and number of years with an FASD diagnosis in Table 4 reflect per 100 individuals screened

### One-way sensitivity analysis

**Fig. 3:**
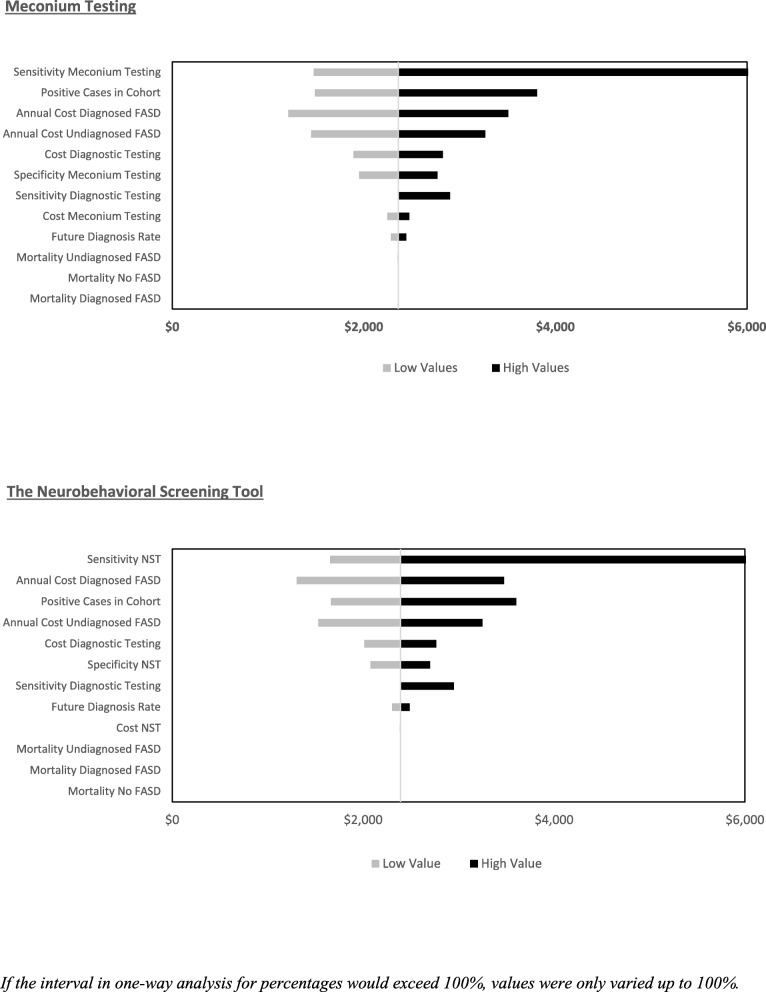
Tornado Plots Meconium Testing and the NST

For both screening strategies, the sensitivity of the screening tool was most impactful on cost-effectiveness. Other impactful parameters included the number of positive cases in the cohort, the annual cost of diagnosed FASD, the annual cost of undiagnosed FASD, and the cost of diagnostic testing. The impact of the cost of screening tools, mortality, and the future diagnosis rate had minimal impact on cost-effectiveness in one-way sensitivity analysis (See Fig. 3).

### Probabilistic analysis

**Fig. 4:**
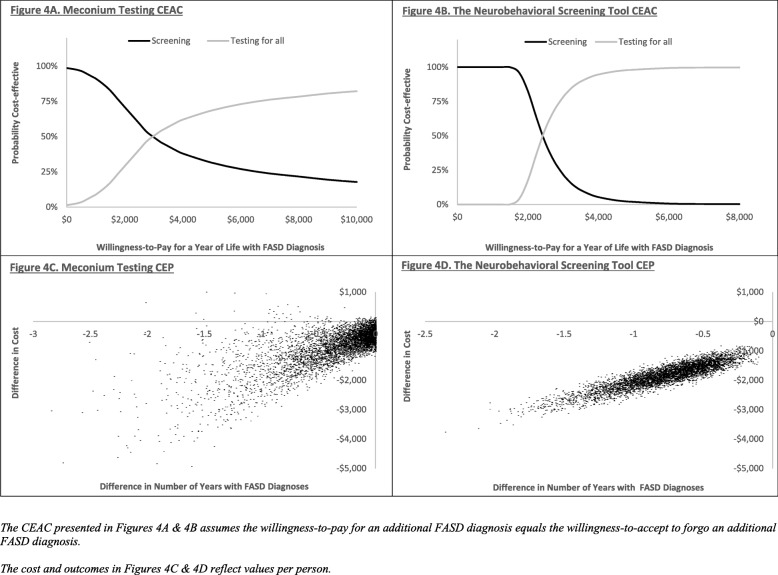
Cost-effectiveness Acceptability Curves and Cost-effectiveness Plane

Using the 5000 ICER generated in PA, a cost-effectiveness acceptability curve (CEAC) was constructed (See Fig. 4a & b). A CEAC uses PA generated ICER to assess the probability that an intervention is cost-effective relative to another intervention across a range of WTP threshold values, dependent on the variability within the model. In the present study, the curve shows the percentage of PA generated ICER that fall below a given WTA threshold for an additional year of life with an FASD diagnosis. At a WTA of $500 there was a 96.5% probability of meconium testing being cost-effective, at $1000 the probability was 91.2%, and by $3000 the probability was less than 50%. At a WTA of $1000, there was a 100% probability of the NST being cost-effective, at $2000 the probability was 82.4%, and by $3000, the probability was well below 50%.

Figure 4c & d show cost-effectiveness planes (CEP). The CEP plots the difference in outcomes on the horizontal axis versus the difference in cost on the vertical axis for each of the 5000 PA generated ICER. The CEP provides a visual representation of an intervention’s likelihood of being cost saving, more effective, and cost-effective relative to another intervention conditional upon the variability in the model.

## Discussion

The present study set out to assess the value for money of tools used to screen children suspected of FASD. By characterizing, the trade-off associated with screening children suspected of FASD prior to diagnostic testing, the present study can provide guidance to physicians and decision-makers evaluating which tools to use and the extent to which FASD screening should be undertaken. Both of the screening tools evaluated resulted in cost savings and fewer diagnosed years of life than a no screening strategy in which all children suspected of FASD receive diagnostic testing. Probabilistic analyses supported this finding. Since diagnosis can be key to children receiving timely and appropriate health and educational services, cost-savings must be weighed against the fewer years of life with a diagnosis associated with screening. Though not directly tested, findings suggest that screening may be an approach to optimize the efficient use of diagnostic resources in jurisdictions where demand for diagnostic testing exceeds supply.

The findings of the present study will have implications for the evaluation of new technologies. At present, there are screening technologies being developed that show promise in identifying patients with FASD [37]. Though these screening tools will enable greater accuracy and testing over the entire lifespan, they will also come at greater cost. Having some understanding regarding current options from a value for money standpoint will be instrumental in making informed decisions regarding the next generation of screening tools.

The present study’s model represents a simplistic approximation of the diagnostic process for FASD and would benefit from additional information. Notably, information on: the optimal criteria for a positive screen for screening tools; the impact of an FASD diagnosis on patients’ HRQoL, use of healthcare services, and mortality; the probability of patients who receive a false negative in screening receiving diagnoses in subsequent years; and the accuracy of diagnostic testing. Furthermore, WTP for an FASD diagnosis is unlikely to be known in most jurisdictions. Future study using cost-utility analysis would be beneficial, as WTP for quality-adjusted life years are better understood in many jurisdictions than WTP for years of life with an FASD diagnosis. The present study was conducted form the perspective of the healthcare system, as FASD is likely to affect the criminal justice and educational systems, broader cost-perspectives may provide additional insights.

Many of the aforementioned limitations are common in modelled CEA of screening tools [21]. Iragorri and Spackman [21] highlight some of the difficulty associated with obtaining cost and outcome data for the undiagnosed populations in CEA of screening procedures and highlight the need for assumptions in these studies. A key driver of the lack of data is that screening can lead to missed diagnoses and studies tend not to be conducted on undiagnosed patient groups, as these patients are difficult to identify.

It should be mentioned that screening children in jurisdictions where there is not diagnostic capacity should not be undertaken. In the event that diagnostic testing is not available, screening should not be used as a substitute for diagnostic testing, as this could lead to misdiagnoses and inappropriate care.

## Conclusions

Screening children suspected of FASD prior to diagnostic testing was associated with cost-savings but fewer years of life with a diagnosis for both screening tools evaluated from the perspective of the healthcare system. The findings of the present study will be of interest to decision-makers investigating which screening tools to use and the extent to which screening in general should be used.

## Supplementary information

**Additional file 1.** Estimated Cost of Undiagnosed FASD.

## Data Availability

For a copy of the present study’s model, please contact the corresponding author PB.

## Abbreviations

CAPHC: Canadian Association of Pediatric Health Centres
CEA: Cost-effectiveness Analysis
CEAC: Cost-effectiveness Acceptability Curve
CEP: Cost-effectiveness Plan
FAEE: Fatty Acid Ethyl Esters
FASD: Fetal Alcohol Spectrum Disorder
ICER: Incremental Cost-effectiveness Ratio
NST: Neurobehavioral Screening Tool
PA: Probabilistic Analysis
QALY: Quality Adjusted Life Years
SD: Standard Deviation
SMR: Standardized Mortality Ratio
WTA: Willingness-to-Accept
WTP: Willingness-to-Pay
