# Supplementary material for: The cost-effectiveness of screening tools used in the diagnosis of fetal alcohol spectrum disorder: a modelled analysis
Source: BMC Public Health. 2019 Dec 27;19:1746. doi: 10.1186/s12889-019-8110-5 (PMC6935188; doi:10.1186/s12889-019-8110-5)
Supplement: Supplementary file 1 — Additional file 1. Estimated Cost of Undiagnosed FASD. [file 12889_2019_8110_MOESM1_ESM.docx]

**Additional file 1. Estimated Cost of Undiagnosed FASD**

**Psychiatrist Services**

(1) Total annual cost for psychiatric services reported by Stade et al., (2006) = $858.65

(2) Number of patients who received an FASD diagnoses directly in McLachlan et al (2015) = 45

- Number of patients recommended to receive psychiatric treatment = 25 (55.6%)

(3) Number of patients whose diagnosis was deferred in McLachlan et al (2015) = 9

- Number of patients recommended to receive psychiatric treatment = 3 (33.3%)

(4) Average cost of psychiatric services in the undiagnosed population:

$858.65 * 45 * (1/25) * 3 * (1/9) = $515.19

(5) Cost reduction in undiagnosed group (2006 $CAD):

$858.65 - $515.19 = $343.46

**Psychologist Services**

(1) Total annual cost for psychiatric services reported by Stade et al., (2006) = $925.00

(2) Number of patients who received an FASD diagnoses directly in McLachlan et al (2015) = 45

- Number of patients recommended to receive psychiatric treatment = 25 (55.6%)

(3) Number of patients whose diagnosis was deferred in McLachlan et al (2015) = 9

- Number of patients recommended to receive psychiatric treatment = 3 (33.3%)

(4) Average cost of psychiatric services in the undiagnosed population:

$925.00 * 45 * (1/25) * 3 * (1/9) = $555.00

(5) Cost reduction in undiagnosed group (2006 $CAD):

$925.00 - $555.00 = $370.00

**Combined Reduction of Cost**

(1) Cost reported by Stade et al., (2006) subtract psychologist and psychiatrist fees:

$3,426 - $343.36 - $370.00 = $2,713

(2) Converted from 2003 CAN$ to 2017 CAN$:

$2,713*1.27 = $3,441
